# The significance of trisomy 7 mosaicism in noninvasive prenatal screening

**DOI:** 10.1186/s40246-019-0201-y

**Published:** 2019-04-11

**Authors:** Yiming Qi, Jiexia Yang, Yaping Hou, Fangfang Guo, Haishan Peng, Dongmei Wang, Qianyi Du, Aihua Yin

**Affiliations:** 1 Prenatal Diagnosis Centre, Guangdong Women and Children Hospital, Guangzhou, 511400 Guangdong China; 2 Maternal and Children Metabolic-Genetic Key Laboratory, Guangdong Women and Children Hospital, Guangzhou, 511400 Guangdong China

**Keywords:** Trisomy 7, Noninvasive prenatal testing (NIPT), Confined placental mosaicism (CPM)

## Abstract

**Background:**

This study was an evaluation of the role of noninvasive prenatal testing (NIPT) in the detection of trisomy 7 in prenatal diagnosis.

**Method:**

A total of 35 consecutive cases underwent screening for trisomies by cell-free DNA testing between April 2015 and November 2017 due to suspicious NIPT results; these cases represented 0.11% of patients (35/31,250) with similar frequencies of abnormal results among the laboratories performing the tests. NIPT was offered to further screen for common fetal chromosomal abnormalities. Karyotype analysis, chromosomal microarray analysis (CMA), and next-generation sequencing (NGS) were used to detect 20, 14, and 25 patients, respectively, who accepted confirmatory diagnostic testing.

**Results:**

High-risk results by NIPT were recorded for trisomy 7 alone in 29 women: dual aneuploidy in 4 patients and multiple aneuploidy in 2 patients. Karyotype analysis of amniotic fluid cells was normal in all 20 pregnancies, suggesting a probability of confined placental mosaicism. Further CMA data were obtained in 14 of the cases mentioned above, and 2 fetuses were detected with positive results with copy number variation. The NGS results suggested that all these samples were placental chimerisms of chromosome 7, except for one sample that was found to be an additional chimerism of chromosome 2, which was also consistent with the NIPT result.

**Conclusion:**

Our results may be useful for the counseling of pregnant women in the detection of trisomy 7 by NIPT.

## Background

Since its introduction in 2011, noninvasive prenatal testing (NIPT) by massively parallel sequencing of cell-free DNA (cfDNA) has profoundly transformed prenatal screening for prenatal aneuploidy. In the latest meta-analysis, the pooled sensitivity was 99.4% for trisomy 21, 97.7% for trisomy 18, and 90.6% for trisomy 13 [1]. The specificity for these aneuploidies was 99.9% to 100% [2]. NIPT has a detection rate of sex chromosome aneuploidies of approximately 90% and a false-positive rate of 1% [3]. Although NIPT can potentially evaluate all 24 chromosomes to identify abnormalities of the pregnant female, placenta, or fetus, current bioinformatics algorithms typically only report on chromosomes 21, 18, 13, X, and Y. The detection of “no target” autosomal aneuploidies has often been a serendipitous finding following diagnostic testing for one of the target autosomal trisomies or for sex chromosome aneuploidies [4]. Thus, the sequencing results from other chromosomes should be masked. The phenotypic consequences of this anomaly are highly variable and may depend on several variables, including the specific chromosome involved, the distribution and proportion of the trisomic line in embryonic and extraembryonic lineages, and the presence of uniparental disomy (UPD) in the euploid cell line [5]. Therefore, the finding of a mosaic autosomal trisomy in amniocytes may require additional cytogenetic and/or molecular work and may leave questions unanswered at the time of post-amniocentesis counseling. Such problems are especially compelling when a rare type of mosaicism is diagnosed.

Currently, a few of the laboratories choose to report this “no target” chromosome testing as a supplemental part of NIPT. In a large cohort study performed recently, NIPT data were generated from 89,817 unique pregnancies, showing that single rare autosomal trisomies (RATs) were the most frequently observed chromosome abnormalities and accounted for 0.34% of the total sample [6]. Among these, trisomy 7 was the most frequently observed RAT [7, 8], followed by trisomies 15, 16, and 22 [5, 9]. In addition, the frequency of trisomy 7, which is the most commonly reported RAT in both the NIPT and chorionic villi sampling (CVS) data sets, was comparable at 0.0746% and 0.0795%, respectively [7].

Unlike whole-chromosome fetal aneuploidy, which mostly results in early fetal loss, the clinical manifestations of autosomal aneuploidies vary widely. Specifically, counseling related to rare autosomal aneuploidies is made difficult by limited case reports and variable expressivity. Confined placental mosaicism (CPM) for chromosomes 8, 15, and 16 has been well described and results in a spectrum of fetal outcomes, from no clinical phenotype to fetal growth restriction [5, 10, 11]. For trisomy 7, most affected conceptions are thought to result in spontaneous abortions during the first trimester. So, a NIPT-positive trisomy 7 conventionally detected after gestational age (GA) probably had some mosaic trisomy 7 in the placenta and/or in the fetus.

In this paper, we report the pregnancy situation and postnatal follow-up data in a series of 35 consecutive cases with single or combined trisomy 7, which were detected by NIPT and referred to our institutions for genetic counseling.

## Methods

### Sample collection

In this study, 35 consecutive cases underwent screening for trisomies by cfDNA testing between April 2015 and November 2017. All patients received detailed pretest counseling and provided written informed consent for the test.

### NIPT

Blood samples from pregnant women were collected for NIPT. From each pregnant woman, 5 mL of peripheral blood was obtained in an ethylene diamine tetra-acetic acid-anticoagulated tube before invasive procedures, and plasma was separated within 8 h following a double-centrifugation protocol. All subsequent procedures, including cfDNA isolation, library construction, and sequencing, were performed according to the instructions of JingXin Fetal Chromosome Aneuploidy (T21, T18, T13) Testing Kits (CFDA registration permit No. 0153400300). Based on our previous study, we developed a technique that uses the read length to estimate the concentration of fetal cfDNA in maternal plasma by sequencing [12]. The fetal DNA concentration was calculated as a quality control procedure, as described in Yin’s paper [12]. Combined GC-correction and *z*-score testing methods were used to identify fetal autosomal aneuploidy for trisomy, as described in Liao’s paper [13]. A *z*-score range from − 3 to 3 was considered to indicate a low risk of a trisomy chromosome, and if *z*-scores were > 3, the sample was in the high-risk zone.

### Karyotyping analysis

Invasive sampling was performed for high-risk cases. The metaphase chromosome G-banding karyotyping was performed at a level of 320 to 400 bands. The results of karyotyping were used as the gold standard. CPM was defined by the detection of the abnormal cell line only by CVS; conversely, true fetal mosaicism (TFM) was defined by the presence of the chromosomal abnormality in both the chorionic villus sample and the amniotic fluid.

### CMA

All follow-up confirmatory diagnostic testing was performed either prenatally (on amniotic fluid) or postnatally on infant blood samples, which included karyotype and chromosomal microarray analysis (CMA). CMA was performed using custom Baylor Genetics oligonucleotide arrays, manufactured by Agilent Technologies, Inc. (Santa Clara, CA), according to the manufacturer’s instructions.

### Placental sequence

The placenta tissue (10 mg) was placed in a sterile vessel under aseptic conditions and was then washed with sterile PBS buffer to facilitate the extraction of DNA. The extracted DNA fragment was interrupted by ultrasonic wave and enzyme repair. A tail and joint were added to construct the sequencing library, and quantitative quality control was carried out on the sample. Multiple sequencing libraries were sequenced with index markers. The sequencing was carried out using the BioelectronSeq 4000 high-throughput sequencing instrument of CapitalBio Technology., Ltd. analytical methods, as described earlier.

### Follow-up

Pregnancy outcome information was gathered from referring doctors and from antenatal and postnatal laboratory results. Additional follow-up data were collected by a telephone interview. The following outcomes were analyzed: delivery, pre- and postnatal growth, psychomotor development, major malformations, and other diseases/complications.

## Results

### Demographic characteristics of pregnant women undergoing NIPT

Seven chromosome aneuploidies were suspected from NIPT results in 0.11% of patients (35/31,250) with similar frequencies of abnormal results among the laboratories performing the tests. The average maternal age and GA were 30.8 years and 20 + 4 weeks, respectively. Nine patients underwent NIPT due to advanced maternal age (> 35 years). A total of 24 patients tested positive on the serum screening test, including 15 with a high risk of fetal T21 (> 1/250), 9 with an intermediate risk (typically 1/250–1/1000), and the other 4 with a single index abnormality. Two patients underwent NIPT screening as a second-line approach after the identification of the widening of the posterior cranial fossa on ultrasound. The nuchal translucency was performed in 35 NIPT-positive cases, and no cases had enlarged thickness; the mean thickness was 1.9 mm (Table 1).

**Table 1:** Maternal characteristics and gestational age of blood sampling

Clinical basic information	Number
Racial	
Han nationality	30
Chuang nationality	4
Unknown	1
Maternal age	30.8
BMI	21.5
Conception of pregnancy	
Naturally conceived	35
Other	0
Fetus number	
Singleton	35
Other	0
Smoking	
Yes	0
None	35
GA at NIPT	20 weeks and 4 days
Average maternal age	9
Pregnancy risk factors	
High risk of T21	15
Intermediate risk of T21	9
Single HCG MOM**↑**	4
HCG MOM**↑**	28
Widening of posterior cranial fossa	2
Threatened premature birth	1
Rh incompatibility	1

**↑**: HCG MOM increase

### Positive NIPT for trisomy 7

High-risk results by NIPT were recorded for trisomy 7 alone in 29 women, dual aneuploidy in 4 patients (trisomy 7/trisomy 2, trisomy 7/trisomy 3, trisomy 7/trisomy 11, and trisomy 7/monosomy X), and multiple aneuploidy in 2 patients (trisomy 7/trisomy 8/trisomy 2 and trisomy 7/trisomy 8/trisomy 3/trisomy 20). The results of the samples from these 35 pregnant women showed that the male phenotype accounted for 62.8% (22/35), slightly more than the female phenotype (34.2%) (12/35). The mean fetal fraction of these subjects was 17.2% (range 7.4–33.0%), and the mean sequence unique read was 4.26 M (range 3.31–5.93 M). The average *z*-score of trisomy 7 was 17.0 (range 5.57–49.43). More than half (25/35) of the women who accepted prenatal diagnostic testing as a follow-up to NIPT chose amniocentesis. No complications according to prenatal diagnostic procedures occurred (Table 2).

**Table 2:** NIPT results of 35 high-risk pregnant women

Case	cfDNA fraction	Reads	*z*-score	Other abnormalities
1	9.1	4.86	12.682	/
2	17.2	3.94	17.21	/
3	16.1	4.69	11.38	/
4	31.8	3.31	49.43	/
5	15.7	4.42	14.811	/
6	18.5	4.61	12.351	/
7	26.1	3.67	16.104	/
8	19.8	3.51	21.037	/
9	10.3	3.99	6.198	T2
10	18.1	5.16	26.462	/
11	11.1	4.19	6.608	/
12	15	3.06	20.423	/
13	16.1	4.19	9.163	/
14	33	3.34	28.678	/
15	16.2	4.59	19.621	/
16	13.8	5.02	5.057	/
17	7.4	5.93	6.051	/
18	24.4	4.47	11.63	/
19	19.4	3.6	27.152	/
20	10.4	4.05	10.44	/
21	12.5	4	10.781	T8/T3/T20 M13/M22
22	15.1	5.28	23.606	XO
23	21	4.45	26.887	/
24	11.6	4.94	21.757	T11
25	25.4	4.75	16.262	T3
26	20.4	4.07	18.185	T8/T2
27	20.9	3.68	29.183	/
28	17.6	4.28	12.686	/
29	11.7	4.45	25.839	/
30	15.9	3.56	22.122	/
31	14.2	4.48	14.649	/
32	11.8	3.64	10.195	/
33	27.5	3.85	15.559	/
34	14.5	4.86	6.557	/
35	11.2	4.78	9.23	/

### Confirmatory diagnostic testing

**Fig. 1:**
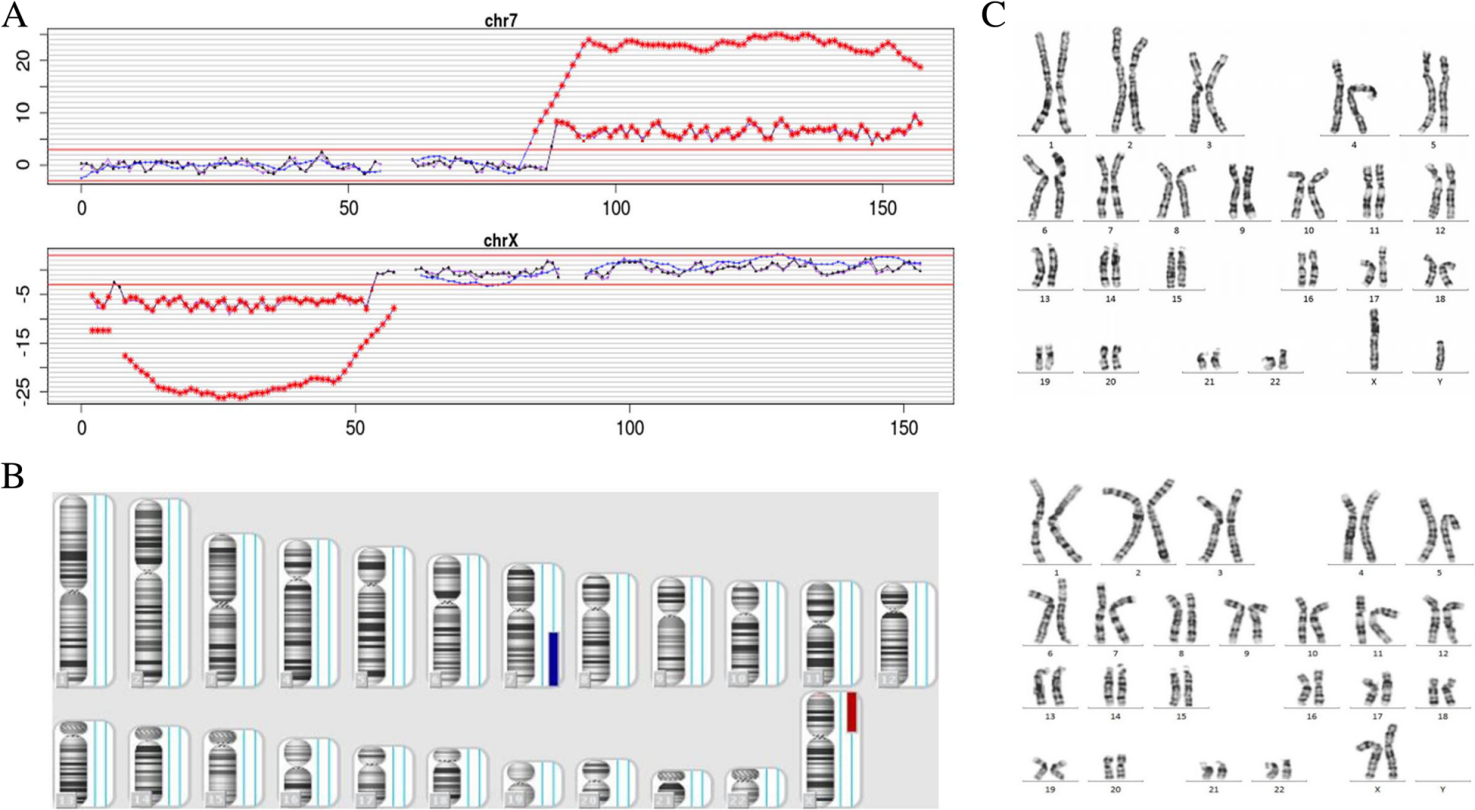
NIPT and CMA results for case 22 and karyotype results for the parents of case 22. **a** NIPT result for case 22. Chr7: dup (90 M–157 M); chrX: del (0.1 M–52 M). **b** CMA result for case 22. The chromosomal regions in the red mark were the missing pieces of that deletion at the position of 7q21.13q36.3(89,040,945-159,119,707), and the blue marks are the missing pieces at the position of Xp22.33p11.22(168,551-53,973,336). **c** Karyotype results for the parents of case 22 were normal

**Fig. 2:**
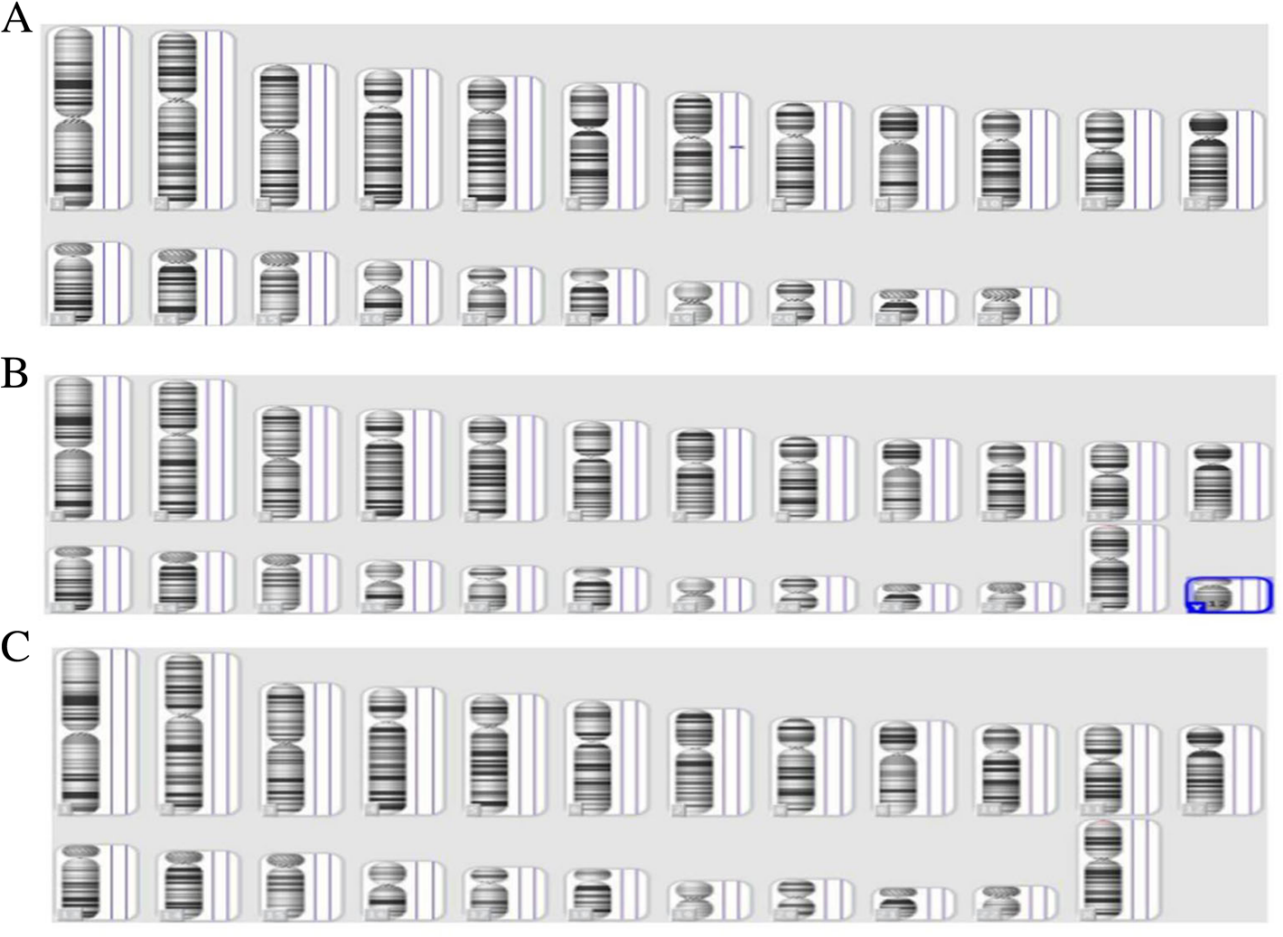
CMA assay results for case 23 and her parents. **a** CMA result for case 23. The chromosomal regions in the red circle were the missing pieces of a novo ~ 1.5 Mb deletion at the position of 7q11.23(72,600,482-74,175,485). **b** CMA result for the father of case 23 were normal. **c** CMA result for the mother of case 23 were normal, too

**Fig. 3:**
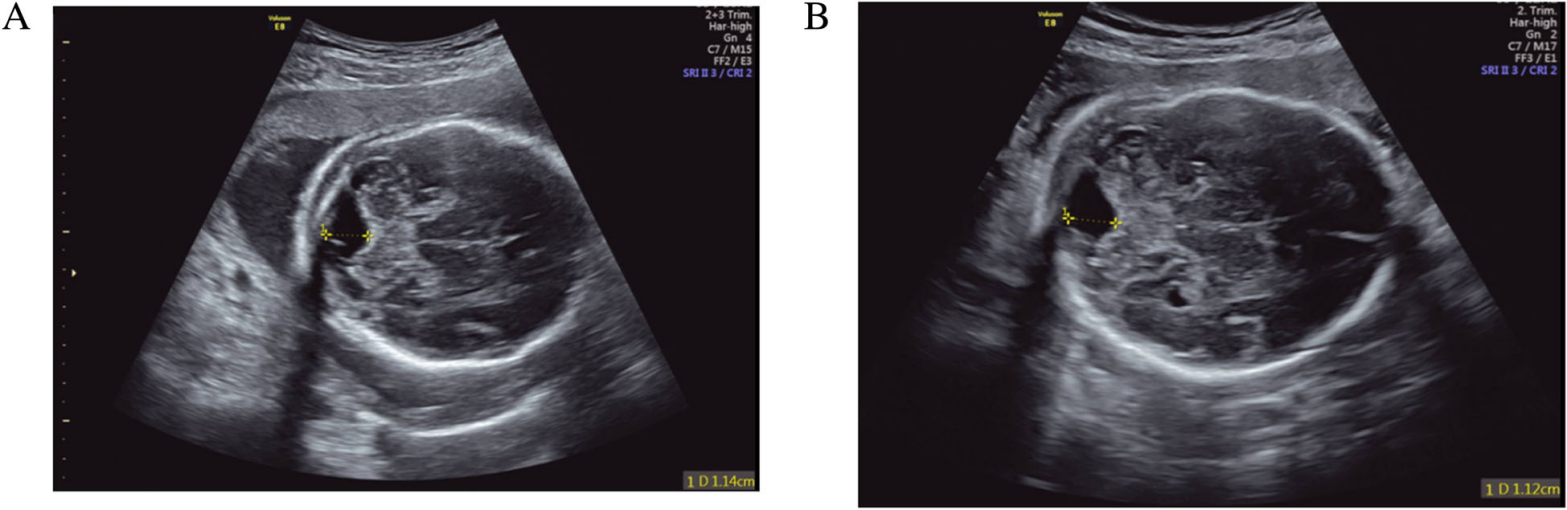
Ultrasound examination images. **a** Ultrasound examination result at 22 weeks. The width of the posterior cranial fossa was 11.4 mm. **b** Ultrasound examination results at 28 weeks. The width of the posterior cranial fossa was 11.2 mm

The karyotype analysis of amniotic fluid cells was normal in all 20 pregnancies, suggesting a probability of CPM. Further CMA data were obtained in 14 of the cases mentioned above (Table 3), and 2 fetuses showed positive results. One of them (case 22) was proven to have a 70.1-Mb duplication at 7q21.13-q36.3, combined with a 53.8-Mb deletion at Xp22.33-p11.22 (Fig. 1b). Taking into account the possibility of balanced translocation, for which the detection performance of NIPT was poor (Fig. 1a), cytogenetic analysis karyotypes of parents were taken, and both parents showed normal results (Fig. 1c). The other case (case 23) revealed a 1.5-Mb duplication at 7q11.23 (Fig. 2a). A CMA of peripheral blood was also performed in both parents (Fig. 2b and c). The results were normal, which might shed light on the finding that the 7q11.23 duplication in the fetus is de novo. A total of 10 placental tissues were retained from the 25 patients who accepted confirmatory diagnostic testing (Table 3). Sequence data suggested that all these samples were placental chimerisms of chromosome 7, except for one, in which an additional chimerism of chromosome 2 was detected. Therefore, it can be inferred that type 1 CPM is involved in 100% of the ten cases (nearly 100% of the NIPT-suspected T7 were of placental origin). Not only did the average chimeric ratio have a wide spectrum, ranging from 15% to 80%, but also for each placental chimeric distribution in six site biopsies (three from the maternal side and three from the fetal side) that were taken for sequencing, a clear difference was shown (Table 3). The ultrasound examination at 22 weeks GA and 28 weeks GA showed that the fetus posterior cranial fossa was 11.4 mm and 11.2 mm, respectively, which was slightly wider than normal (Fig. 3).

**Table 3:** The NIPT results verified by Karyotype analysis, CMA, and next-generation sequencing (NGS)

Case	Karyotype analysis	CMA	NGS
1	/	/	/
2	46,XN	NA	/
3	46,XN	NA	/
4	46,XN	NA	47,XX,+ 7 [80]/46,XX [20]
5	/	/	/
6	/	/	/
7	/	/	/
8	46,XN	NA	47,XX,+ 7 [20]/46,XX[80]
9	46,XN	NA	48,XX, + 2, + 7 [15]/46,XX[85]
10	46,XN	NA	/
11	/	/	/
12	46,XN	NA	47,XY,+ 7 [70]/46,XY [30]
13	46,XN	NA	47,XX,+ 7 [30]/46,XX[70]
14	46,XN	NA	/
15	46,XN	NA	/
16	/	/	/
17	46,XN	NA	/
18	/	/	/
19	46,XN	NA	/
20	46,XN	NA	47,XX,+ 7 [40]/46,XX[60]
21	46,XN	NA	/
22	46,XO	7q21.13q36.3 (89,040,945-159,119,707) × 3/Xp22.33p11.22 (168,551-53,973,366) × 1	/
23	46,XN	7q11.23 (72,600,482-74,175,485) × 3	/
24	46,XN	NA	47,XX,+ 7 [15]/46,XX[85]
25	46,XN	NA	/
26	46,XN	NA	/
27	/	/	/
28	46,XN	NA	47,XX,+ 7 [65]/46,XX [35]
29	46,XN	NA	47,XX,+ 7 [20]/46,XX[80]
30	46,XY	NA	/
31	46,XN	NA	/
32	/	/	/
33	46,XN	NA	/
34	/	/	/
35	46,XN	NA	47,XX,+ 7 [15]/46,XX[85]

*CMA* chromosomal microarray analysis, *NGS* next-generation sequencing, *NA* normal

### Pregnancy outcomes

Complications for pregnant women included cesarean delivery (*n* = 9), gestational hypertension (*n* = 2), gestational diabetes mellitus (*n* = 2), and placental abruption (*n* = 1). Two patients elected to terminate their pregnancies based on NIPT predictions of suspicious multiple aneuploidy with T7/T8/T3/T20 and CMA results with 1.5-Mb duplication at 7q11.23. The suspected T7/T8/T2 patient was the same patient with threatened abortion who experienced the complication of spontaneous abortion 2 weeks after NIPT at 16 weeks GA. Twenty-nine out of the 35 pregnancies had been successfully followed up for 6 months to 2 years. Three children were born with low birthweight (< 3rd percentile), and 3 underwent preterm delivery with an average GA of 36 weeks. No children required neonatal intensive care unit (NICU) admission. Among the 2 pregnancies in which CNV was detected, only one case (case 22) displayed macrocephaly and discordance of limbs with body size at birth. Case 22 was born at 37 weeks + 4 days and had a femur length of 65 mm and a humerus length of 56 mm. All of the remaining patients delivered healthy children with normal postnatal physical and psychomotor development, and no congenital anomalies required surgical correction.

Details of NIPT findings, the results of cytogenetic assessment, placental sequence, and pregnancy outcomes for all cases of abnormal chromosome 7 suspected after NIPT are listed in Table 1.

## Discussion

### Incidence of T7 CPM

It has been reported that autosomal trisomies were the most frequently detected mosaic abnormalities in chorionic villi (CV), and the incidence rate was approximately 54.5%. On the other hand, autosomal trisomies showed the lowest risk of fetal involvement (7.5%) [7]. The principal mechanism of formation of the abnormal mosaic cell line is the nondisjunction error (either meiotic or mitotic), which gives rise to an aneuploid cell line; thus, most autosomal trisomies remain confined to the placenta, as they are almost universally lethal, either in a homogeneous or in a mosaic form, when affecting the fetus [14]. Therefore, rare trisomies in prenatal diagnosis samples are common findings in products of conception [15–18].

Trisomy 7 is one of the most common aneuploidies detected by CVS [7, 8, 19, 20]. Nonmosaic trisomy 7 is thought to result in spontaneous abortions during the first trimester in most conceptions [9, 19]. Ayub et al. [3] substantiates the hypothesis that CPM 7 most frequently occurs due to somatic duplication; therefore, the risk of fetal UPD 7 is low. The single case of fetal UPD 7 identified in this study showed fetal intrauterine growth retardation (IUGR), as described in the literature, and the origin of CPM was determined to be meiotic. Here, we also showed that trisomy 7 suspected upon NIPT accounted for 0.11% of the total patients (35/31,250), which was similar to the frequencies found among the laboratories performing the tests.

### T7 detected by NIPT

It is certainly possible that the major trisomy 7 detected by NIPT has varying levels of CPM, as cfDNA comes from the villi of placenta, which is always obtained after 12 weeks GA. However, NIPT still needs to be combined with other analyses to allow for the identification of the origin of CPM and for a more reliable prediction of pregnancy outcomes [21]. It is known that fetal cell-free DNA in maternal peripheral blood originates from trophoblasts and mainly consists of placental DNA. That is, NIPT is a test used to analyze placenta-derived DNA, allowing secondary results that reflect the pathology of the *z*-score [12]. There are a variety of factors that affect NIPT outcomes, such as placental chimerism, maternal obesity, and maternal cancer [22]. Among these 35 NIPT cases positive for T7, 14 cases had a high risk of fetal T21, 8 cases had an intermediate risk, and 2 cases had a human chorionic gonadotropin (HCG) pregnancy test abnormality. As would be expected, most of the T7 patients revealed serological marker abnormalities, including AFP, UE3, and HCG, which are secreted by the placenta. Further observations revealed that almost all the cases had an substantially higher MoM for HCG. It is speculated that sustained proliferation of the mutant trisomy 7 cell line in the trophoblast leads to a continuous secretion of HCG, which is heavily weighted in the calculation of screening risk. In a review of the literature, most of the T7 results were unexpected findings of other abnormal serological screening results. We could find that abnormalities in serological parameters often occur in other RATs, so this may not be unique to T7 or other 7 abnormalities; thus, the presence of publication bias should be considered.

There is a direct positive relationship between the concentration of HCG and placenta. The larger the placenta or the more energetic the cells were, the more HCG might have been secreted. Chromosomes 7 contain significant regions of imprinting, which may have contributed to altered gene expression and possibly to morphological alterations suggestive of a partial hydatidiform mole [23]. Although it is possible that both the specific chromosomes involved and the dosage (number) of extra paternal chromosomes may act in concert to influence villous morphology, one study found that the parental origin of trisomy for only chromosome 7 did not affect the placental morphology. Reddy et al. [8] suggested that the random mutant trisomy 7 cell line in the placenta may suffer no functional compromise, and trisomy 7 cells may continue to proliferate up to a particular threshold ratio of normal cells to trisomic cells. Additionally, Yong PJ et al. [24] also suggested that when a pregnancy with trisomy was confined to the placenta, the placental weight was always reduced (with a normal feto-placental weight ratio), although the level of placental trisomy was not correlated with placental weight. Thus, trisomy may alter placental function rather than have a direct hypoplastic effect on placental growth. However, more in-depth studies beyond routine pathology are required to identify how trisomy affects placental function.

The cffDNA originates from apoptotic trophoblastic cells [25], and the placenta sheds a large amount of cffDNA into maternal circulation. Thus, the result from NIPT shows the karyotype of uncultured placental cells. As reported, the average fetal fraction in the maternal plasma is 10% to 15% when measured between 10 and 20 gestational weeks. Therefore, the concentration of cffDNA in this CPM 7 group was not significantly different from that in the normal population, which was 17.8% of the mean fetal fraction tested in this study. It might also shed light on the conclusion that trisomy 7 cells alter placental function rather than have a hypoplastic effect.

### T7 CPM and multi-CPM combination

Fetal death in utero may occur due to the high percentage of trisomy 7 cells in the placenta or the presence of trisomy 7 cells in the fetus [7]. Hsu et al. [26] reported a case with 100% trisomy 7 cells in the villi from the placenta, which ultimately terminated in fetal death. More frequently, no dysmorphic features were described in the literature. In this study, the chimeric ratio in the placenta ranged from 15 to 80, and no stillbirth, abortion, or severe complications occurred in any of the 10 cases. This finding offered some indication of the features that generally had a good prognosis in the absence of TPM, although the proportion of chimerism is as high as 80 in this study. Chimeric embryos have self-correcting mechanisms, which may arise according to the following theories: (1) the dominant growth of euploid cells; (2) the self-correction of abnormal cells; and (3) the aggregation of normal cells to the inner cell mass [27, 28]. Trisomic cell populations can be self-corrected by delaying anaphase or by not separating chromosomes to lose excess chromosomes, but these situations are unlikely because single parent diploids are rarely found in blastocysts [29]. It was found that aneuploidy cells grew slowly, died during apoptosis, and gradually decreased in number during embryonic development; eventually, healthy fetuses were born. The number of chromosomes generated by chimerism was not related to the success rate of embryonic development. Except for chromosome 17 [30], almost all chromosomes with chimera had a normal pregnancy.

To date, mosaicism affecting two chromosomes has not been described in the largest study of rare trisomy mosaicism. In the case of trisomy, more than two chromosomes could have a larger impact on placental function than either trisomy alone, which may contribute to fetal demise. Ultimately, the process of abnormal chromosomal development may also affect the fetus. Studies [10, 15, 31] have also reported that specific chromosomal trisomies have been observed in CPM more frequently than others, with the trisomy of chromosomes 7, 16, and 18 being the most prevalent. When CPM was superimposed with T7, T2/T3/T8/T11/T20 seemed more common than T16/T18 [9]. However, when the other or multiple CPMs simultaneously existed, regardless of the percentage of trisomy 7 cells, it was difficult to predict the prognosis of the fetus. In this study, when CPM was superimposed with T8 and T2, spontaneous abortion inevitably occurred during the second trimester; however, when CPM was combined with single T2 or T3 or T11, no aberrants were detected except for the FTS. If this event was accompanied by monosomy X, a series of clinical phenotypes, such as macrocephaly and discordance of limbs with body size at birth, would be evident. However, this event finally confirmed the real reason for the decrease in the dose of chromosome X, which was expressed a form of monosomy X in NIPT caused by the affected fetus.

### Confirmation test by amniocentesis

It should be noted that the trisomy 7 mosaicism detected by NIPT is relatively common and rarely confirmed at amniocentesis. The confirmation test was karyotype analysis of amniotic fluid cells for all 25 informed consent pregnancies. On the one hand, when NIPT had been performed and until the results were provided, almost all the women were more than 14 weeks GA, and this period is no longer suitable for CVS [19]. On the other hand, which is much more important, as the exact embryo-fetal developmental stage in which the mitotic error occurs cannot be established with certainty with karyotyping, the retrieval of a mosaic in CV does not necessarily imply fetal involvement (true fetal mosaicism or TFM), as it could be restricted to placental CPM. For this reason, in this situation, a confirmatory karyotype on amniocytes is recommended to discriminate a generalized mosaicism (placenta and fetus affected by the abnormal cell line) from a confined mosaicism (only the placenta was affected but not the fetus). Amniotic fluid sampling is considered the standard procedure of confirmation because it provides cells for cytogenetic analysis coming mainly from fetal anatomical districts, including the urogenital tract, the respiratory apparatus, and the epithelial system representing different embryological layers [32]. Karyotype analysis of amniotic fluid cells was normal in all of the tested pregnancies, insinuating a minimal possibility of TFM.

### Prognosis

Nearly all of the suspected trisomy 7 subjects had good perinatal outcomes, and no obvious anomalies were detected. Case 22 was proven to have a 70.1-Mb duplication at 7q21.13-q36.3 combined with a 53.8-Mb deletion at Xp22.33-p11.22. Due to a lack of prenatal ultrasound monitoring, macrocephaly and discordance of limbs with body size at birth were found. Chromosome Xp22.33-p11.22 deletions involve several phenotypes, such as autism, chondrodysplasia punctata, craniofrontonasal dysplasia, ichthyosis, and heterocellular hereditary persistence of fetal hemoglobin [33–36]. In this case, we could not find that the child had the above results. Chromosome 7q36 deletions and duplications are rare genomic disorders that have been reported in a limited number of children with developmental delays, growth retardation, and congenital malformations. Altered dosages of SHH and HLXB9, both located in 7q36.3, are believed to play roles in the phenotypes associated with these rearrangements. Al Dhaibani et al. [37] reported a chromosome 7q36.1-q36.2 triplication in a child with IUGR complications prenatally and with developmental delay, distinctive facial features, and multiple congenital complications postnatally. The increased GALNT11 dosage potentially alters the Notch signaling pathway, explaining the pathogenicity of 7q36 triplication [37]. Upon querying the database, we believed that the phenotype of this case was consistent with chromosome 7q21.13-q36.3 duplications. It is necessary to study further cases with similar genomic rearrangements to make final conclusions about the pathogenicity of this triplication. In this study, the parents of case 22 insisted on having this baby after the eugenics genetic counseling, which suggested that the fetus may have a birth defect.

Case 23 revealed a 1.5-Mb duplication at 7q11.23, the area of which included 32 genes and was classified as VOUS. However, the fetus showed a normal phenotype both prenatally and postnatally. To date, this location has not been previously described as a pathogenic area. Kivirikko et al. [8] reviewed 17 reported cases of trisomy 7 mosaicism, diagnosed both prenatally and postnatally, and suggested that a 50% risk of abnormalities should be given to parents when this mosaicism was diagnosed prenatally. However, they failed to take into account the ascertainment bias introduced by the consideration of case reports, which will overrepresent abnormal cases and postnatally diagnosed cases. In our opinion, it is necessary to perform more tests, such as cytogenetic analysis, if the prenatal results are abnormal. Although T7 was the most frequent autosomal mosaic trisomy in CV, it was mostly confined to the placental tissue rather than affecting the fetus.

In addition to microdeletion/microduplication, fetal UPD, which is associated with specific clinically determined phenotypes for some chromosomes, is another important risk factor for malformation. As the UPD does not involve an increase in the dose of chromosome, NIPT often performs poorly in detecting it. Fortunately, it has been reported that CPM 7 is most frequently the result of mitotic misdivision [21]; therefore, the risk of fetal UPD 7 is also extremely low [38]. Consistent conclusions have been drawn from further studies, and no UPD was detected among the 25 cases with the CMA test. Therefore, when trisomy 7 cells are only confined to the placenta, they do not cause obvious abnormalities. Once the trisomy cells exceeded the placenta, UPD or TPM might be present; some of these patients will suffer IUGR, kidney, or cardiovascular abnormalities prenatally. Thus, once a T7 has been inadvertently detected by NIPT, ultrasound detection of a fetus that is small for gestational age and/or that has renal abnormalities should be carefully assessed by serial sonography. If there are negative findings under ultrasound scanning, further puncture examination should be considered to provide more information, especially for those “precious fetuses”, for whom the invasive detection has the potential to result in unnecessary diagnostic procedures and unnecessary pregnancy termination procedures. Conversely, if an IUGR/VSD/hydronephrosis or other issue had already been discovered by ultrasound, and if NIPT meanwhile showed a case that was positive for trisomy 7, then under these circumstances, amniocentesis after 16 weeks would be necessary to rule out the possibility of a trisomy 7 fetus.

Our study provides further evidence that this anomaly, when detected by NIPT only, is associated with an overall good prognosis; in fact, most of the pregnancies resulted in the birth of healthy infants with normal birth weight and normal postnatal physical and psychomotor development. Therefore, even if low-level mosaicism cannot be excluded, it is unlikely that growth and psychomotor development anomalies are associated with it.

## Conclusion

In conclusion, our results may be useful for the counseling of pregnant women when trisomy 7 is detected by NIPT. Nevertheless, the prenatal detection of trisomy 7 is challenging for genetic counseling, and it is often difficult to predict the associated postnatal phenotype. Our data showed an overall positive prognosis for cases with an apparent CPM, and no obvious anomalies were detected by fetal ultrasound examinations. Therefore, larger studies are warranted to better define the associated risk of forward neurodevelopmental anomalies, psychomotor development, or other postnatal complications.

### Shortcomings

The postnatal follow-up of live-born infants was too short to detect anomalies in psychomotor development or other future complications.

## Abbreviations

cfDNA: Cell-free DNA
CMA: Chromosomal microarray analysis
CNV: Copy number variation
CPM: Confined placental mosaicism
CVS: Chorionic villi sampling
NGS: Next-generation sequencing
NICU: Neonatal intensive care unit
NIPT: Noninvasive prenatal testing
RATs: Rare autosomal trisomies
TFM: True fetal mosaicism
UPD: Uniparental disomy

## References

[1] Gil MM, Akolekar R, Quezada MS (2015). Analysis of cell-free DNA in maternal blood in screening for aneuploidies: meta-analysis. Ultrasound Obstet Gynecol.

[2] Mackie FL, Hemming K, Allen S (2017). The accuracy of cell-free fetal DNA-based non-invasive prenatal testing in singleton pregnancies: a systematic review and bivariate meta-analysis. Bjog.

[3] Ayub S, Gadji M, Krabchi K (2016). Three new cases of terminal deletion of the long arm of chromosome 7 and literature review to correlate genotype and phenotype manifestations. Am J Med Genet A.

[4] Kornman L, Palmadias R, Nisbet D, et al. Non-invasive prenatal testing for sex chromosome aneuploidy in routine clinical practice. Fetal Diagn Ther. 2017. 10.1159/000479460 28873375

[5] Kotzot D, Utermann G (2005). Uniparental disomy (UPD) other than 15: phenotypes and bibliography updated. Am J Med Genet A.

[6] Pertile MD, Halks-Miller M, Flowers N (2017). Rare autosomal trisomies, revealed by maternal plasma DNA sequencing, suggest increased risk of feto-placental disease. Sci Transl Med.

[7] Bilimoria KY, Rothenberg JM (2003). Prenatal diagnosis of a trisomy 7/maternal uniparental heterodisomy 7 mosaic fetus. Am J Med Genet.

[8] Kivirikko S, Salonen R, Salo A (2002). Prenatally detected trisomy 7 mosaicism in a dysmorphic child. Prenat Diagn.

[9] Zaragoza MV, Millie E, Redline RW (1998). Studies of non-disjunction in trisomies 2, 7, 15, and 22: does the parental origin of trisomy influence placental morphology?. J Med Genet.

[10] Udayakumar AM, Al-Kindy A (2013). Constitutional trisomy 8 mosaicism syndrome: case report and review. J Pediatr Genet.

[11] Su MT, Liang YL, Chen JC (2013). Non-mosaic uniparental trisomy 16 presenting with asplenia syndrome and placental abruption: a case report and literature review. European Journal of Medical Genetics.

[12] AH Y, CF P, X Z (2015). Noninvasive detection of fetal subchromosomal abnormalities by semiconductor sequencing of maternal plasma DNA. Proc Natl Acad Sci U S A.

[13] Liao C, Yin AH, Peng CF (2014). Noninvasive prenatal diagnosis of common aneuploidies by semiconductor sequencing. Proc Natl Acad Sci U S A.

[14] Sachdev NM, Maxwell SM, Besser AG (2017). Diagnosis and clinical management of embryonic mosaicism. Fertility & Sterility.

[15] Chen CP, Lin HM, Su YN (2012). Mosaic trisomy 9 at amniocentesis: prenatal diagnosis and molecular genetic analyses. Taiwanese Journal of Obstetrics & Gynecology.

[16] Chen CP, Su YN, Su JW (2013). Mosaic trisomy 12 at amniocentesis: prenatal diagnosis and molecular genetic analysis. Taiwan J Obstet Gynecol.

[17] Genuardi M, Tozzi C, Pomponi MG (1999). Mosaic trisomy 17 in amniocytes: phenotypic outcome, tissue distribution, and uniparental disomy studies. Eur J Hum Genet.

[18] Boghossian NS, Hansen NI, Bell EF (2014). Mortality and morbidity of VLBW infants with trisomy 13 or trisomy 18. Pediatrics.

[19] Reddy KS, Blakemore KJ, Stetten G (1990). The significance of trisomy 7 mosaicism in chorionic villus cultures. Prenat Diagn.

[20] Tchirikov M, Merinsky A, Strohner M (2010). Prenatal diagnosis of a recombinant chromosome 7 resulting in trisomy 7q11.22 → qter. Am J Med Genet A.

[21] Kalousek DK, Langlois S, Robinson WP (1996). Trisomy 7 CVS mosaicism: pregnancy outcome, placental and DNA analysis in 14 cases. Am J Med Genet.

[22] Yaron Y (2016). The implications of non-invasive prenatal testing failures: a review of an under-discussed phenomenon. Prenat Diagn.

[23] Norris-Kirby A, Hagenkord JM, Kshirsagar MP (2010). Abnormal villous morphology associated with triple trisomy of paternal origin. J Mol Diagn.

[24] Langlois S, Yong PJ, Yong SL (2006). Postnatal follow-up of prenatally diagnosed trisomy 16 mosaicism. Prenat Diagn.

[25] Norton ME, Jacobsson B, Swamy GK (2015). Cell-free DNA analysis for noninvasive examination of trisomy. N Engl J Med.

[26] Hsu LY, Yu MT, Neu RL (2015). Rare trisomy mosaicism diagnosed in amniocytes, involving an autosome other than chromosomes 13, 18, 20, and 21: karyotype/phenotype correlations. Prenat Diagn.

[27] Helen B, Graham SJL, Niels VDA (2016). Mouse model of chromosome mosaicism reveals lineage-specific depletion of aneuploid cells and normal developmental potential. Nat Commun.

[28] Munné S, Blazek J, Large M (2017). Detailed investigation into the cytogenetic constitution and pregnancy outcome of replacing mosaic blastocysts detected with the use of high-resolution next-generation sequencing. Fertility & Sterility.

[29] Fragouli E, Alfarawati S, Spath K (2017). Analysis of implantation and ongoing pregnancy rates following the transfer of mosaic diploid-aneuploid blastocysts. Hum Genet.

[30] Bazrgar M, Gourabi H, Valojerdi MR (2013). Self-correction of chromosomal abnormalities in human preimplantation embryos and embryonic stem cells. Stem Cells Dev.

[31] Armstrong AA, Gaw SL, Platt LD (2018). Mosaic trisomies 8, 9, and 16.

[32] Grati Francesca Romana, Malvestiti Francesca, Branca Lara, Agrati Cristina, Maggi Federico, Simoni Giuseppe (2017). Chromosomal mosaicism in the fetoplacental unit. Best Practice & Research Clinical Obstetrics & Gynaecology.

[33] Zumwalt J, Moorhead C, Golkar L (2012). Fourteen-month-old girl with facial skin thinning.

[34] Chang YC, Smith KD, Moore RD (1995). An analysis of fetal hemoglobin variation in sickle cell disease: the relative contributions of the X-linked factor, beta-globin haplotypes, alpha-globin gene number, gender, and age. Blood.

[35] Kofman-Alfaro SH, Vaca ALJ, Cuevas-Covarrubias SA (2000). A novel partial deletion of exons 2–10 of the STS gene in recessive X-linked ichthyosis. J Investig Dermatol.

[36] Twigg SRF, Christian B, Elzen MEP, Den V (2013). Cellular interference in craniofrontonasal syndrome: males mosaic for mutations in the X-linked EFNB1 gene are more severely affected than true hemizygo tes. Hum Mol Genet.

[37] Dhaibani MAA, Allingham-Hawkins D, El-Hattab AW (2017). De novo chromosome 7q36.1q36.2 triplication in a child with developmental delay, growth failure, distinctive facial features, and multiple congenital anomalies: a case report. Bmc Medical Genetics.

[38] Warburton D (2002). Trisomy 7 mosaicism: prognosis after prenatal diagnosis. Prenat Diagn.

